# Functional Connectivity Is Altered in Concussed Adolescent Athletes Despite Medical Clearance to Return to Play: A Preliminary Report

**DOI:** 10.3389/fneur.2016.00116

**Published:** 2016-07-25

**Authors:** Mary R. Newsome, Xiaoqi Li, Xiaodi Lin, Elisabeth A. Wilde, Summer Ott, Brian Biekman, Jill V. Hunter, Pramod K. Dash, Brian A. Taylor, Harvey S. Levin

**Affiliations:** ^1^Department of Physical Medicine and Rehabilitation, Baylor College of Medicine, Houston, TX, USA; ^2^Department of Neurology, Baylor College of Medicine, Houston, TX, USA; ^3^Department of Radiology, Baylor College of Medicine, Houston, TX, USA; ^4^Department of Orthopedic Surgery, UTHealth McGovern Medical School, Houston, TX, USA; ^5^Department of Radiology, Texas Children’s Hospital, Houston, TX, USA; ^6^Department of Neurobiology and Anatomy, UTHealth McGovern Medical School, Houston, TX, USA

**Keywords:** sports concussion, functional connectivity, adolescent, memory, athletic injuries, traumatic brain injury

## Abstract

Recovery following sports-related concussion (SRC) is slower and often more complicated in young adolescent athletes than in collegiate players. Further, the clinical decision to return to play is currently based on symptoms and cognitive performance without direct knowledge of brain function. We tested the hypothesis that brain functional connectivity (FC) would be aberrant in recently concussed, asymptomatic athletes who had been cleared to return to play. A seed-based FC analysis measured the FC of the default mode network (DMN) (seeds = anterior cingulate cortex, posterior cingulate cortex (PCC), right lateral parietal cortex, and left lateral parietal cortex) 30 days after SRC in asymptomatic high school athletes cleared to return to play (*n* = 13) and was compared to the FC of high school athletes with orthopedic injury (OI) (*n* = 13). The SRC group demonstrated greater FC than the OI group between the PCC and the ventral lateral prefrontal cortex, as well as between the right lateral parietal cortex and lateral temporal cortex (with regions both outside of and within the DMN). Additionally, the OI group demonstrated greater FC than the SRC group between right lateral parietal cortex and supramarginal gyrus. When relating the FC results to verbal memory performance approximately 1 week and 1 month after injury, significantly different between-group relations were found for the posterior cingulate and right lateral parietal cortex seeds. However, the groups did not differ in verbal memory at 1 month. We suggest that changes in FC are apparent 1-month post-SRC despite resolution of post-concussion symptoms and recovery of cognitive performance in adolescent athletes cleared to return to play.

## Introduction

In comparison with sports-related concussion (SRC) in professional and collegiate athletes, imaging studies of concussed high school athletes are sparse. This gap is concerning in view of evidence that recovery is slower and more often complicated in young adolescent athletes as compared with their collegiate counterparts (1, 2). The few imaging studies of SRC and of playing a season of football in high school athletes have reported effects on both structural and functional connectivity (FC) (3, 4). A recent diffusion tensor imaging (DTI) study found that cumulative exposure to subconcussive head impacts over a single season in 12 high school football players without concussion was negatively related to the structural integrity of white matter (WM) tracts, whereas similar changes were not present in a control group (3). Resting state functional magnetic resonance imaging (rsfMRI) has disclosed altered FC in the default mode network (DMN) and in the executive function and attention networks in symptomatic high-school athletes who were imaged within 2-months post-concussion. Borich et al. (4) found increased posterior cingulate cortex (PCC) connectivity and decreased connectivity in the frontal and parietal cortices within the DMN. Within the executive function network, Borich et al. (4) observed increased connectivity in the right frontal pole in the concussed group, as compared with the control group. These investigators also found that within the ventral attention network, the concussed players had increased activity in the left frontal operculum cortex.

Abbas et al. (5) employed rsfMRI to image non-concussed high school football players prior to, during, and post-season. Using the posterior cingulate/precuneus as a seed, the number of connections with other brain regions was increased in the contact athletes in months 2 and 3, but reduced in months 1 and 4, suggesting both long-term effects of cumulative exposure (e.g., change from preseason to month 1) and variability related to short-term changes associated with head impacts (months 2 and 3) even in the absence of concussion. Abbas et al. (5) attributed the increased FC to compensatory, alternative connections as a result of head impacts sustained over several years prior to the study. Variability across imaging sessions in the number of DMN connections during the season was greater in the contact athlete group, suggesting short-term effects of head impacts. Taken together, the imaging studies to date in high school athletes support the possibility that the effects of SRC on functional and structural brain connectivity persist beyond the date when players become asymptomatic and are cleared to return to play. This discordance between recovery defined by consensus clinical criteria and resolution of altered brain imaging has given rise to a debate on management of SRC (6). One of the gaps in studies of high school athletes is analyzing FC at a time that is temporally contiguous to recovery from SRC and return to play according to consensus guidelines (1).

We conducted a pilot study of FC in concussed high-school athletes whom we imaged at approximately 1-month post-injury after their post-concussion symptoms (PCS) had resolved and they had been cleared to return to play. Based on findings in patients imaged after sustaining a mild traumatic brain injury (mTBI) (7), we hypothesized that FC in the DMN would be altered, whereas connections with lateral prefrontal cortex would be increased on rsfMRI. In view of a meta-analysis supporting the sensitivity of verbal learning and memory to SRC, the typical recovery within 14 days post-injury, and the clinical relevance of this cognitive function in high school students (2, 8), we also studied its relation to FC at 1 month post-injury.

## Materials and Methods

### Design

This prospective, dual cohort observational study evaluated alteration of brain FC in asymptomatic high-school athletes who had been cleared for return to play by 1 month post-SRC. We compared FC of regions in the DMN of concussed athletes imaged at about 1 month post-injury with data from adolescent players who had sustained an OI. As explained above, we focused the regression analysis on the relation of FC to verbal learning and memory.

### Subjects

We studied 13 adolescent athletes (Table 1) who sustained SRC, which was defined according to consensus guidelines, i.e., “transient disturbance of brain function due to a sports-related blow to the head or other body region” causing acceleration of the head followed by onset of PCS, including physical (e.g., headache), cognitive (e.g., confusion/poor attention), and emotional (e.g., depression, anxiety) domains (1). The definition also included loss of consciousness (LOC) (if present) less than 30 min and post-traumatic amnesia (PTA) duration of less than 24 h (1). One athlete in the SRC group had a brief LOC. Resolution of PCS, recovery of cognitive performance on Immediate Post-Concussion Assessment and Cognitive Testing (ImPACT) (9) to preseason level, and clearance by a licensed health provider to return to play by day 30 post-injury were also inclusion criteria. All mTBI subjects were recruited from the Ironman Sports Medicine Institute, Houston, TX, USA and underwent in-person clinical evaluations of PCS and cognition within 3 days post-injury, between days 7 and 10, and on day 30 or 31. All SRC subjects were scanned within 37 days post-injury to ensure that the window for imaging closely followed return to play.

**Table 1:** **Mean (SD) demographic characteristics for the SRC and OI control groups**.

	SRC	OI controls	*p*
Age (years)	16.0 (1.1)	16.4 (1.3)	0.6004
Gender	1 F; 12 M	6 F; 7 M	0.0730
Ethnicity/Race	3 AA; 0 A; 6 H; 4 C; 0 Other	5 AA; 1 A; 3 H; 3 C; 1 Other	0.5568
Contact sports	12 Yes, 1 No	9 Yes, 4 No	0.3217

*A, Asian; AA, African American; C, Caucasian; F, female; H, Hispanic; M, male*.

For comparison, 13 adolescent athletes who were also 13–19 years old and participated in a sport but who had been orthopedically injured (OI) served as controls. The orthopedic injuries in the OI group were also mild. Similar to the criterion for concomitant extracranial injury severity in the SRC group (see below), extracranial injury worse than mild (Abbreviated Injury Scale ≥2) (10) was an exclusion for athletes in the OI group. One athlete in the OI group had a previous concussion. The assessment and imaging protocol for the OI group was identical to the procedures used in the SRC group. However, the post-injury interval for imaging differed between groups, SRC: 33.1 days (±1.7); OI: 124.7 days (±69.0). The intervals for recruitment and imaging of athletes with OI were longer because we have found that their imaging findings are stable between the first week and 3 months after OI (11) and thus less time sensitive (11).

Exclusions for the SRC group included acute neurologic deterioration to a Glasgow Coma Scale (12) score <13, neurosurgical intervention, abnormal computed tomographic (CT) scan, concomitant extracranial injury worse than mild (Abbreviated Injury Scale ≥2) (10), pre-injury conditions, which confound effects of SRC (e.g., epilepsy, schizophrenia, bipolar illness, mental deficiency, hospitalization for TBI). The rationale for exclusion based on an abnormal CT was that this imaging modality would have been performed within 24 h after injury, usually because of persistent or progressive impairment of consciousness indicating a brain injury worse than the definition of SRC (1). Pathology seen on the 30-day MRI was not a basis for exclusion because this imaging was performed for research purposes long after the acute phase when neurologic deterioration or neurosurgical evacuation of a mass lesion would have occurred. In fact, no athlete in this study had evidence of neurologic deterioration that would have prompted a clinical CT and excluded the athlete from this study. Exclusion criteria for both groups included substance dependence and inability to speak fluent English. Subjects who exhibited motion greater than 2 mm translation and 2° rotation or inability to complete the scan (e.g., claustrophobia) were excluded.

Groups did not significantly differ in age, race, ethnicity, or handedness (all *p*’s > 0.10). There tended to be fewer females in the concussed group than in the OI group, Fisher’s Exact Test *p* = 0.0730, because fewer females were treated for SRC at the recruitment site during the study period. All subjects were interviewed and examined by a clinician who is experienced in the evaluation of SRC and did not have access to the subject’s imaging data. The study was approved by the Institutional Review Board at University of Texas Health Science Center at Houston and Baylor College of Medicine. Informed consent was obtained for each subject.

### Assessment of Memory

Memory was evaluated on the Day 7–10 and on Day 30 visits using the Hopkins Verbal Learning Test-Revised (HVLT-R) (13). In this standard measure of episodic memory, the examiner verbally presents the same list of 12 words on each of three trials and instructs the participant to recall the words in any order. Twenty-five minutes later, the examiner asks the participant to recall the word list. Total recall summed over the three learning trials and the number of words recalled after the delay are analyzed.

### Image Data Acquisition

Imaging Acquisition. Whole brain imaging was performed using a 32-channel head coil on a Siemens MAGNETOM Trio 3T system. Blood oxygen level dependent (BOLD) T2* weighted echo-planar images (EPI) were acquired as 245 volumes with 40 axial slices of 3.0 mm thickness with a 0.3 mm gap, using a 240 mm field of view (FOV), 70 × 70 matrix, repetition time (TR) of 2030 ms, echo time (TE) of 28 ms, and a 90° flip angle. A set of 3D high-resolution T1-weighted images were also acquired in 176 sagittal slices of 1.2 mm thickness with 256 mm FOV, 240 × 256 matrix, TR of 2300 ms, TE of 2.96 ms, and an 9.0° flip angle.

## Statistical Analysis

### Assessment of Memory

Two-tailed *t*-tests for independent groups were performed to evaluate group differences. Because of the small sample sizes and preliminary nature of the study, effect sizes (Cohen’s *d*) were calculated to further characterize group differences, where *d* = 0.2 is a small effect, *d* = 0.5 is a moderate effect, and *d* = 0.8 is a large effect.

### Functional Connectivity Image Processing and Analysis

The Functional Connectivity Toolbox (Conn) (14) within SPM8 (Wellcome Department of Cognitive Neurology, University College, London, UK) implemented in Matlab (Mathworks Inc., Sherborn, MA, USA) was used to process and analyze data. Functional images of each subject were realigned, co-registered with each subject’s high-resolution anatomical image, normalized to the Montreal Neurological Institute (MNI) template, and smoothed using a 6 mm Full Width – Half Maximum Gaussian filter. Anatomical landmarks in the normalized high-resolution anatomical and functional data were visually checked and compared against the MNI template for each subject. Each subject’s anatomical image was segmented into gray matter, WM, and cerebrospinal fluid (CSF) masks. Physiological noise was addressed by using WM and CSF masks as covariates. Realignment parameters and their first-order derivatives were also covaried. The Artifact Detection Toolbox (14) was used to repair artifact due to frame-by-frame head movement, i.e., scrub (15), and correct global drift. Outlier time points were defined as exceeding three SD from the mean image intensity of the complete resting state run. Outliers were included as regressors in the first level general linear model along with motion parameters. Data were band-pass filtered between 0.008 and 0.09 Hz, the default frequency range in the SPM Conn toolbox. The high-pass value was selected to approximate both SPM’s default value (0.0078 Hz) and a 2-min value suggested as a standard (0.0083 Hz) (16). The low-pass value approximates the frequently reported 0.08 and 0.10 Hz values and SPM’s hemodynamic response function cutoff frequency of.091 Hz. FC was measured with single seeds in the following regions of the DMN: (1) medial prefrontal cortex (MPFC), (2) PCC, (3) left lateral parietal lobe (LLP); (4) right lateral parietal lobe (RLP) (17). Seeds were made available by the Conn software package and were 10-mm spheres centered around the following MNI coordinates MPFC −1 49 −5; PCC −6 −52 40; LLP −46 −70 36; RLP: 46 −70 36.

A general linear model was used to estimate the correlation between the seeds and the whole brain on a voxelwise level for individual participants (first level). Pearson correlation coefficients were then transformed into *z*-scores using Fisher’s method followed by group (second level) random effects analyses. For group analyses, *t*-tests were calculated to investigate whole brain differences in FC between SRC and OI control subjects. Significance was defined by voxel (height) threshold *p* < 0.001 and cluster threshold *p* < 0.05. False Discovery Rate (FDR) corrected for multiple comparisons across the whole brain. The FDR method was further used to correct for the number of tests (four seeds × two tails = 8) in the FC analysis. To relate FC to delayed verbal recall, Spearman correlations between the Fisher-transformed *z*-scores of significant clusters and the HVLT-R delayed and total scores collected at the 7–10 and 30-day post-injury intervals were computed for each group. As in the FC-only analysis, FDR was applied to control for type I error when conducting multiple tests on group differences in the correlations between FC and verbal memory (18).

## Results

### Verbal Learning and Memory

As shown in Table 2, moderate effect sizes were found for group differences in both HVLT-R delayed and HVLT-R total scores at day 7–10. However, by day 30 effect sizes were small to moderate.

**Table 2:** **Mean verbal learning and memory scores for the SRC and OI control groups**.

Tests	Occasion	SRC			OI controls			*p*-value	Cohen’s *d*^a^
		Mean	SD	Range	Mean	SD	Range		
HVLT-R delay	Day 7–10	7.7	1.5	6–10	8.9	1.9	6–12	0.078	0.72 (1.70)
	Day 30	8.8	1.6	5–11	9.3	1.7	6–11	0.486	0.28 (1.66)
HVLT-R total	Day 7–10	23.3	2.3	19–27	25.2	3.9	17–31	0.135	0.61 (3.17)
	Day 30	25.5	3.9	16–32	26.9	2.9	20–30	0.286	0.43 (3.42)

*^a^Cohen’s d effect size 0.2 = small, 0.5 = moderate, 0.8 = large. Pooled standard deviations for the effect sizes are in parentheses*.

*HVLT-R, Hopkins Verbal Learning Test – Revised*.

### Functional Connectivity

#### Medial Prefrontal Cortex

Functional connectivity from the MPFC did not significantly differ between the SRC and OI groups.

#### Posterior Cingulate Cortex

**Figure 1:**
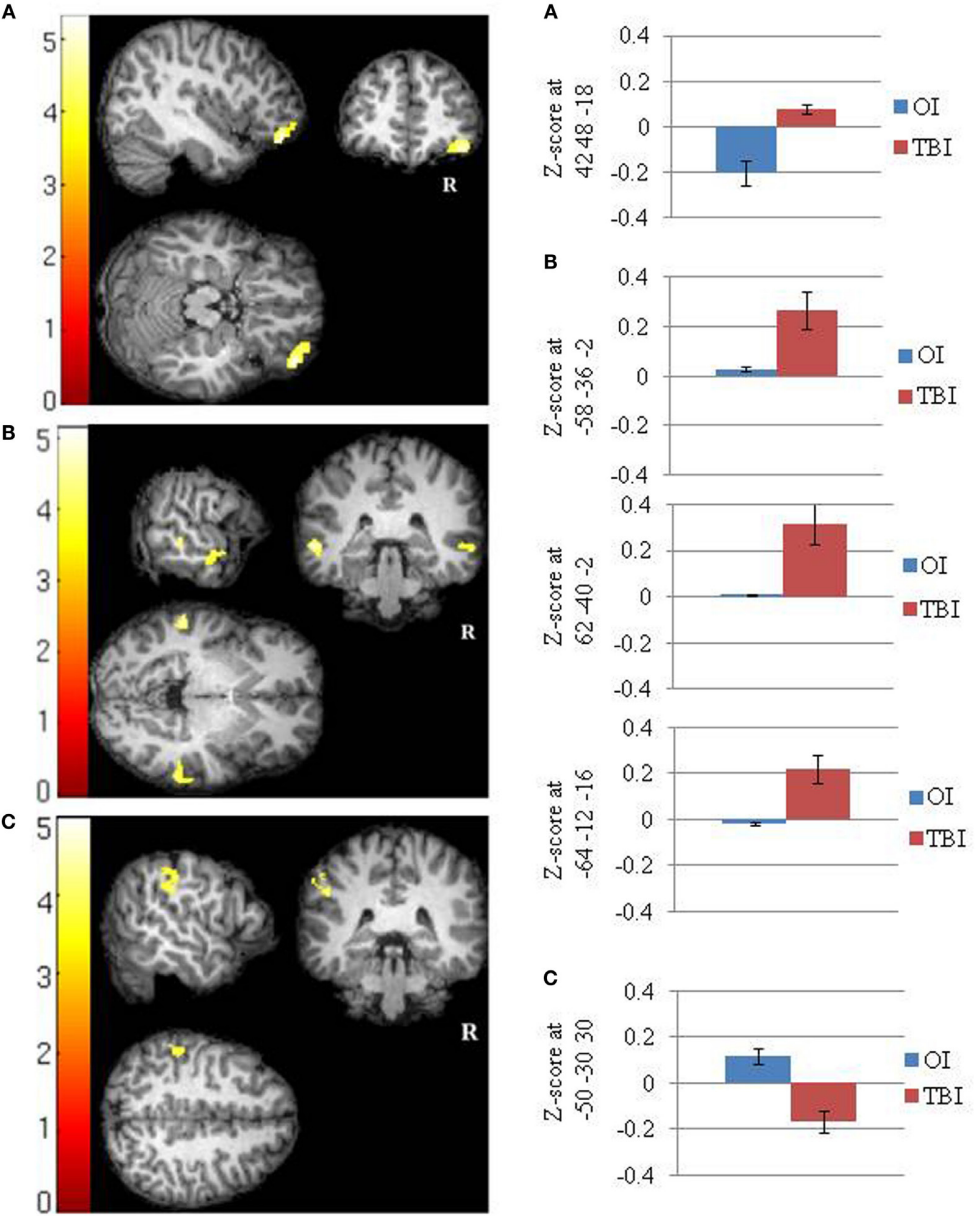
**Significant between groups differences in the functional connectivity of the default mode network in (A) the posterior cingulate cortex (SRC > OI), (B) the right lateral parietal cortex (SRC > OI), and (C) the right lateral parietal cortex (OI > SRC)**. Mean Fisher transformed *z*-scores and their standard errors associated with each cluster are also depicted. Right side of brain is on right side of image.

The SRC group demonstrated greater positive FC than the OI group between the PCC and a cluster (42 48 −18) that included the right ventral lateral prefrontal cortex (Cohen’s *d* = 2.08). The OI group did not demonstrate any greater FC than the SRC group (Figure 1A; Table 3).

**Table 3:** **Between groups *T*-tests showing significant group differences in functional connectivity between the clusters shown and seeds in the (a) posterior cingulate cortex (SRC > OI); (b) right lateral parietal (SRC > OI); and (c) right lateral parietal (OI > SRC)**.

Cluster-level *p* value (corrected)^a^	Cluster size (k)^b^	Most significant coordinates^c^ (*x y z*)	Location
**a. Posterior cingulate cortex**			
SRC > OI			
0.000007	240	42 48 −18	R ventral lateral prefrontal cortex
**b. Right lateral parietal**			
SRC > OI			
0.001988	122	−58 −36 −02	L middle temporal gyrus
0.004590	94	62 −40 −02	R middle temporal gyrus
			R superior temporal gyrus
0.026291	59	−64 −12 −16	L superior temporal gyrus
**c. Right lateral parietal**			
OI > SRC			
0.000008	245	−50 −30 30	L anterior supramarginal gyrus
			L postcentral gyrus

*^a^Probability at the cluster level of significance after random field theory family wise error correction over the whole brain search volume*.

*^b^Number of voxels within a cluster*.

*^c^Negative values along the x-axis are defined to be in the subject’s left hemisphere*.

#### Left Lateral Parietal Lobe

Functional connectivity from the LLP did not significantly differ between the SRC group and the OI controls.

#### Right Lateral Parietal Lobe

The SRC group demonstrated greater positive FC than the OI group between the RLP and three clusters (−58 −36 −02; 62 −40 −02; −64 −12 −16) that included the lateral temporal cortex (i.e., left middle temporal gyrus, right middle temporal gyrus, and right superior temporal gyrus) (Cohen’s *d*s = 1.96, 2.03, 1.83, respectively). The OI group demonstrated greater FC than the SRC group in one cluster composed primarily of the left supramarginal gyrus (−50 −30 30) (Cohen’s *d* = 2.04) (Figures 1B,C; Table 3).

### Correlation of FC with Verbal Learning and Memory

**Figure 2:**
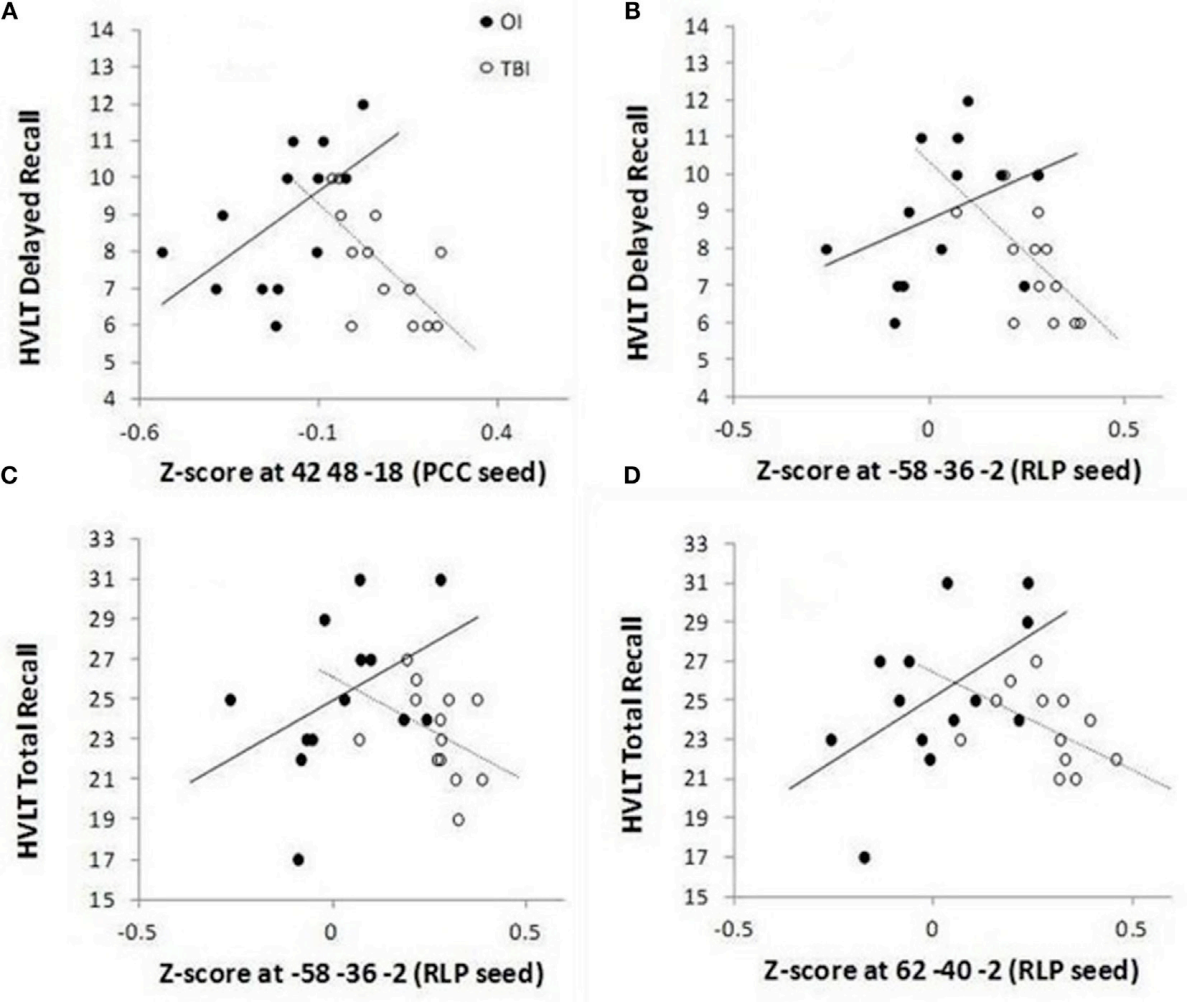
**Group differences for significant correlations between HVLT-R and functional connectivity between the DMN (PCC seed) and ventral lateral prefrontal cortex (A) and between the DMN (RLP seed) and lateral temporal cortex within the DMN (B–D)**. The mean number of words recalled is plotted along the *Y*-axis for delayed recall (recall without presentation of the words on a single trial 25 min after the three learning trials) [**(A,B)**] and for total recall (sum of words recalled immediately after presentation on each of the three learning trials) [**(C,D)**].

To investigate the relation of the five clusters to delayed memory performance, FDR correction was applied to 20 tests. Figure 2 indicates that groups significantly differed on the correlations between the HVLT-R delayed recall 7–10 days after injury and (1) FC between the PCC seed and ventral lateral prefrontal cortex (*p* = 0.00017) and (2) FC between the right lateral parietal lobe and the left middle temporal gyrus (*p* = 0.00288). Similarly, groups significantly differed on the correlations between the HVLT-R total recall 7–10 days after injury and (1) FC between the right lateral parietal lobe and left middle temporal gyrus and (2) FC between the right lateral parietal lobe and right middle temporal gyrus (*p* = 0.00802 and 0.00477, respectively).

Specifically, for the PCC seed, the OI group demonstrated a positive relation between the ventral lateral prefrontal cortex cluster (found to have greater FC in the SRC group than in the OI group in the PCC *t*-test) and HVLT-R delayed and total scores, while the SRC group demonstrated negative relations (see Table 4). For significant relations between clusters associated with the RLP seed and HVLT-R, the relations for the SRC group were also negative, and all the relations for the OI group were positive (Table 4).

**Table 4:** **Within group Spearman correlations between functional connectivity and memory outcomes on HVLT-R**.

HVLT-R memory outcomes	Occasion	Group	Spearman correlation coefficients (*p*-values) (seed/focus of cluster)				
			PCC/VLPFC	RLP/SMG	RLP/L MTG	RLP/RMTG	RLP/L STG
Total recall	1 Week	SRC	−0.483^a^ (0.095)	0.305 (0.311)	−0.522^a^ (0.068)	−**0.619 (0.024)**	0.108 (0.725)
		OI	0.487^a^ (0.092)	−0.294 (0.330)	0.542^a^ (0.056)	0.492 (0.087)	0.219 (0.473)
	1 Month	SRC	−0.470 (0.105)	−0.040 (0.897)	−0.034 (0.912)	−0.359 (0.229)	−0.288 (0.341)
		OI	−0.053 (0.863)	0.212 (0.487)	0.269 (0.374)	0.051 (0.870)	0.373 (0.209)
Delayed recall	1 Week	SRC	−**0.656 (0.015)**	0.124 (0.687)	−**0.647 (0.017)**	0.051 (0.870)	−0.059 (0.848)
		OI	**0.716 (0.006)**	−0.173 (0.572)	0.510^a^ (0.075)	0.320 (0.286)	0.281 (0.352)
	1 Month	SRC	−0.507^a^ (0.077)	−0.105 (0.732)	0.028 (0.926)	−0.399 (0.177)	−0.418 (0.155)
		OI	0.237 (0.436)	0.437 (0.136)	0.299 (0.321)	−0.006 (0.985)	0.322 (0.284)

*The bold correlation coefficients had p-value < 0.05*.

*^a^had p-value between 0.05 and 0.1*.

*L MTG, left middle temporal gyrus; PCC, posterior cingulate cortex; RLP, right lateral parietal; R MTG, right middle temporal gyrus; SMG, supramarginal gyrus; SRC, sports-related concussion; OI, orthopedically injured*.

## Discussion

### Overview

This study sought to address whether previously reported pathophysiological changes in FC resulting from concussion or mild TBI may persist in high school athletes with SRC, despite return-to-play clearance based upon current clinical guidelines. To address this, we examined the DMN utilizing rsfMRI in asymptomatic athletes with SRC at 1-month post-concussion as well as in a comparison group of athletes without SRC, but with OI. We also sought to probe the functional relevance of these changes through examination of the relation of FC with performance on a widely used clinical measure of verbal memory performance. Significant alterations in FC were noted in the athletes with SRC as compared to the comparison group, and a divergent pattern of correlation between FC and memory functioning was observed in the athletes with SRC.

### Verbal Learning and Memory

We found a moderate effect size for group difference on performance of tasks related to verbal learning and memory as measured by the HVLT-R within the day 7–10 window following SRC as compared with non-concussed athletes. This finding is consistent with a meta-analysis showing that verbal learning and delayed recall are especially sensitive to residual effects of SRC (8). However, performance by the SRC group at approximately day 30 no longer differed from the OI comparison group. As described below, the relation of FC to verbal learning and memory in the SRC group was significant in the early period of recovery (e.g., during the day 7–10 post-injury interval), but not after a longer interval following resolution of the memory symptoms.

### Functional Connectivity

Despite the resolution of symptoms and recovered neuropsychological performance in high school athletes who were cleared to return to play within 30 days after SRC, their FC in specific regions measured from rsfMRI differed from the OI group at 30 days post-injury as discussed below.

#### PCC and Ventral Lateral Prefrontal Cortex

The SRC group demonstrated greater FC than the OI group between the PCC and the ventral lateral prefrontal cortex, whereas the athletes who sustained an OI showed the typical anti-correlation found in healthy subjects (17). The increased FC between the PCC and prefrontal cortex in the SRC group is generally consistent with other seed-based analysis reports of subacute concussion or mild TBI. For example, adults with mild TBI of various causes demonstrated increased FC between the PCC and dorsal lateral prefrontal cortex in a seed-based analysis within 24 h of injury (19). At a longer post-injury interval, Mayer et al. (7) found increased FC between the DMN (albeit with the anterior cingulate cortex as opposed to the PCC) and ventral lateral prefrontal cortex in adults with mild TBI at 3–5 months after injury. More recently, Risen and colleagues (20) examined a group of children aged 11–17 years with mild to moderate TBI (half of which were injured as a result of sport-related activity) at an average of 68 days after injury (range 29–102 days) and reported increased FC between the DMN (using the mean time course of three seeds, including the PCC) and premotor cortex.

However, the design of our pilot study differs from these studies by focusing on SRC in asymptomatic high school athletes who met clinical consensus guidelines for return to play prior to imaging approximately 30 days post-injury. Our study also differs from the Risen et al. study in that all of our participants were injured as a result of SRC, had a generally shorter mean post-injury interval, and included a comparison group of non-concussed high school athletes, rather than a group of typically-developing children, thus controlling for pre-injury factors. Although asymptomatic status was not a criterion for recruitment in any of these studies, their finding of altered FC is consistent with our results. In collegiate athletes who were scanned within 24 h of injury and who were asymptomatic, Johnson and colleagues (21) found increased FC with the MPFC, rather than the PCC. Because athletes in the present study were scanned ≈30 days post-injury, our PCC finding may reflect changes in alteration in FC over time, and developmental changes due to age may also impact outcome.

#### Right Lateral Parietal Cortex and Lateral Temporal Cortex

The SRC group also demonstrated increased FC between the right lateral parietal cortex and lateral temporal cortex, a region of the DMN (22). Thus, concussed adolescent athletes demonstrated increased FC with regions both outside and within the DMN (see below).

#### Right Lateral Parietal Cortex and Supramarginal Gyrus

In contrast to the increased FC in the SRC participants for some brain regions relative to the OI group, the SRC group demonstrated decreased FC between right lateral parietal cortex and supramarginal gyrus. The supramarginal gyrus has been implicated in pain (23), which presumably was greater in the athletes with OI; however, results may also have been driven by an anticorrelation in the SRC group.

#### Correlation of FC with Verbal Learning and Memory

When relating clusters that showed group differences in FC to performance on the HVLT-R measured on day 7–10, groups significantly differed on the relations in clusters in which the SRC group demonstrated greater FC than the OI group (the ventral lateral prefrontal cortex cluster associated with the PCC seed, and clusters containing bilateral middle temporal gyri associated with the RLP seed).

#### FC between PCC and Ventral Lateral Prefrontal Cortex

For the cluster that demonstrated greater FC between the PCC and ventral lateral prefrontal cortex, the OI group demonstrated a positive relation with delayed and total recall on the HVLT-R, whereas the SRC group demonstrated a negative relation. Inspection of the individual *z*-scores representing FC (Figure 2) suggests an interesting pattern: although the OI group tended to have slightly better performance on the HVLT-R, both groups were similar in that they tended to perform better when FC z-scores were closer to zero, i.e., when there was no or little (positive or negative) FC between PCC and ventral lateral prefrontal cortex. In the OI subjects (who demonstrated negative z-scores), the positive relation between accuracy rates on the HVLT-R and FC between the PCC and ventral lateral prefrontal cortex suggests that the OI subjects who performed better on the memory measures at 7–10 days had DMN FC that was mildly anticorrelated (e.g., had negative – scores that approached zero) with the task-related network. In the SRC subjects (who had primarily positive *z* scores), the negative relation they demonstrated also suggests that successful memory was associated with *z*-scores closer to zero. Thus, although the groups significantly differed in the comparisons of their relations between FC and HVLT-R, there is a shared pattern wherein good performance in both groups is associated with mild anticorrelations, in the case of the OI group, or mild positive correlation, in the case of the SRC group, between the PCC and task-related network measured 3 weeks later. Stronger positive associations between the DMN and ventral lateral prefrontal cortex may be associated with interference from increased autobiographical and other thought associated with the DMN during cognitive tasks. Such an account would be consistent with the claim that increased FC between the two regions may account for the distractibility and cognitive fatigue associated with traumatic brain injury (7).

By 1 month, no differences in the relation between DMN alteration and memory performance were observed. We attribute the lack of a relation at 1 month to improved verbal memory in the SRC group whose scores on HVLT-R did not differ from those of the OI group.

#### FC between the RLP and Lateral Temporal Cortex

However, a different pattern between performance and FC was observed when the clusters fell within the DMN (i.e., group differences were found in relations between HVLT-R and FC between the RLP and clusters in lateral temporal cortex). To understand this, we again looked at the within group correlation patterns. The OI group demonstrated no special pattern, and correlations between the clusters and HVLT-R in the OI group were not significant. However, the SRC group demonstrated a significant negative relation, suggesting that SRC subjects who performed more poorly on the HVLT-R also showed greater FC between two regions of the DMN.

Bilateral lateral temporal lobes of the DMN are associated with processing autobiographical memory and thinking about the thoughts of others (24). Again, increased internal thought may interfere with the ability to use memory and the attention required to remember. Lateral temporal lobes are also a common area of damage in TBI (25), and potentially their increased involvement is related to injury. Increased FC in a region susceptible to TBI may result from compensation of healthy tissue proximal to tissue that is damaged and is consistent with the increased FC found in lateral frontal lobes, also susceptible to damage in TBI, reported here and in Mayer et al. (7).

### Limitations

Limitations of this study include small sample sizes with limited power to detect group differences, a trend toward greater number of females in the OI group, and longer post-injury interval in the OI group. Small sample sizes are associated with a greater chance of false negatives, reduced likelihood of detecting true positives, and potential overestimation of the magnitude of effects (26–28). The trend of greater number of females in the OI group was a result of fewer females evaluated at the recruitment site who were participants in contact sports. Additionally, we note that a greater number of prospectively recruited female athletes did not participate in the imaging procedures as they were still symptomatic at 1-month post-injury. We also acknowledge that the single occasion for imaging precludes analysis of long-term changes in FC in relation to outcomes. While it was sometimes unavoidable to scan OI patients after the 30-day window, all SRC subjects were scanned by 37 days post-injury. Future analysis will also consider the relation between FC and tasks that may be more sensitive to processing speed and response latency variability. Although we acknowledge that many of the athletes in the SRC group also sustained repetitive, subconcussive head impacts over the course of the season, the trajectory of cognitive recovery and resolution of symptoms supports our attribution of their initial findings to SRC which was diagnosed according to clinical consensus guidelines (1) by a licensed healthcare practitioner.

## Conclusion

We found that asymptomatic high school athletes cleared to return to play within 30 days post-SRC nonetheless had lingering alterations in FC at day 30. Future research may investigate whether delaying return to play in asymptomatic, concussed adolescent athletes with altered FC affects long-term neurobehavioral outcomes. However, at present, there is lack of sufficient research findings to advocate an evidenced-based approach for using imaging to guide return to play.

## Author Contributions

Conceptualized paper (MN, Xiaoqi Li, and HL), acquired (EW, BB, and MN), analyzed (MN, Xiaodi Lin, and Xiaoqi Li) and interpreted (MN, Xiaoqi Li, and HL) data; drafted (MN, Xiaoqi Li, and HL) and critically revised (MN, Xiaoqi Li, Xiaodi Lin, EW, SO, BB, JH, PD, BT, and HL) paper; gave final approval (MN, Xiaoqi Li, Xiaodi Lin, EW, SO, BB, JH, PD, BT, and HL), and agree to be accountable for all aspects of the work (MN, Xiaoqi Li, Xiaodi Lin, EW, SO, BB, JH, PD, BT, and HL).

## Conflict of Interest Statement

The authors declare that the research was conducted in the absence of any commercial or financial relationships that could be construed as a potential conflict of interest.
